# DNA Methylation at Birth Showing Age-Specific Association with Atopy in Children: A Prospective Longitudinal Study

**DOI:** 10.3390/epigenomes10020033

**Published:** 2026-05-27

**Authors:** Nahid Sultana, Fen Yang, Negusse Kitaba, Stephen Potter, John W. Holloway, S. Hasan Arshad, Hongmei Zhang

**Affiliations:** 1 Division of Epidemiology, Biostatistics, and Environmental Health, School of Public Health, University of Memphis, Memphis, TN 38152, USA; 2 Human Development and Health, Faculty of Medicine, University of Southampton, Southampton SO16 6YD, UK; 3 The David Hide Asthma and Allergy Research Centre, Isle of Wight P030 5TG, UK; 4 Clinical and Experimental Sciences, Faculty of Medicine, University of Southampton, Southampton SO16 6YD, UK

**Keywords:** childhood atopy, DNA methylation, epigenetics, gene expression, methQTL, skin prick test (SPT)

## Abstract

**Background**: The relationship between neonatal DNA methylation (DNAm) and childhood atopy, particularly its temporal dynamics, remains inadequately characterized. Establishing this will provide insights into the epigenetic mechanisms underlying atopy development. **Methods**: Skin prick tests (SPT) for 11 common allergens were performed in the Isle of Wight third-generation birth cohort (IOWF2) at ages 1, 3, and 6 years. Atopy was defined as a positive response to one or more allergens on SPT. DNAm at birth at 294,265 CpGs from umbilical cords (*n* = 192) or Guthrie cards (*n* = 107) was screened (through R package *ttscreening*) for potential association with atopy. Pathway enrichment analysis of screened CpGs was performed using the *missMethyl* package in R. Associations between CpGs that passed screening and atopy status were assessed via logistic regressions with repeated measures, adjusting for age, sex, and birth weight. Age-specific associations were examined via DNAm × age interactions. CpGs showing age-specific association were further tested in the parental cohort (IOWBC (F1); *n* = 717). Multiple testing was controlled using FDR-adjusted *p*-values at 0.05. **Results**: In total, 601 CpGs passed screening. Pathway enrichment analysis identified enrichment of the cell activation pathway (GO:0001775; FDR-adjusted *p*-value = 0.017). DNAm at 502 CpGs in F2 showed age-specific associations with atopy. Among these, 102 CpGs showed consistent directions in F1, and 14 were statistically significant (*p*-value < 0.05). Except for cg01519508 (*FOXF1*), DNAm–atopy associations weakened over time at the remaining 13 CpGs. **Conclusions**: At certain CpGs, DNAm at birth is associated with childhood atopy in an age-dependent manner, and for CpGs showing association at an earlier age, such associations weaken at later ages.

## 1. Introduction

Atopy is an immunoglobulin E (IgE) mediated immune response to common environmental allergens that often emerges early in life, predisposing individuals to chronic allergic conditions such as asthma, eczema, and allergic rhinitis [1,2]. According to the Centers for Disease Control and Prevention (CDC), approximately one in five children in the United States experiences seasonal allergies, and 12.7% children have eczema [3].

Despite its increasing prevalence, the etiology of atopy remains unclear due to its complex biological mechanisms. Emerging evidence suggests that gene–environment interactions play an essential role in shaping immune development and subsequent allergic responses later in life [4,5,6]. One such mechanism is DNA methylation (DNAm), an epigenetic modification involving the addition of a methyl group to a cytosine–phosphate–guanine (CpG) dinucleotide, which regulates gene expression without altering the underlying DNA sequence [7]. Numerous studies have investigated the association between DNA methylation and childhood atopy and the development of atopic diseases [8,9,10,11,12]. Some of these studies focused on infants with atopy assessed by the skin prick test [9,11], while others assessed associations at later ages, e.g., during adolescence [12]. Almost all these studies assessed the associations concurrently. In addition, DNA methylation in these studies was measured postnatally, and postnatal assessment of DNAm can be affected by reverse causation. From the perspective of the relationship between early age DNAm and later atopic status, utilizing DNAm at birth would be more informative and diminishes the concern of reverse causation. However, to our knowledge, no studies have investigated whether DNAm at birth is associated with childhood atopy, and to what extent such associations change as a child grows.

In this study, we investigated whether DNAm patterns measured at birth were associated with atopy, as assessed by skin prick testing (SPT), at various stages in early childhood. Specifically, we examined epigenome-wide DNAm at birth in relation to sensitization to 11 common allergens, including food, indoor, and outdoor aeroallergens. We hypothesized that DNAm at birth is associated with subsequent atopy in an age-specific manner. Furthermore, to evaluate the reproducibility of our findings, we conducted replication analyses in the parental cohort.

## 2. Results

### 2.1. Descriptive Statistics

The demographic characteristics and SPT status of children from the complete third-generation cohort (*n* = 611) and sub-cohort (*n* = 299) are shown in Table 1. The subsample was representative of the complete cohort, as demonstrated by *p*-values > 0.05 for the variables included in our analyses.

### 2.2. Findings in the Discovery Cohort (IOWF2)

**Figure 1:**
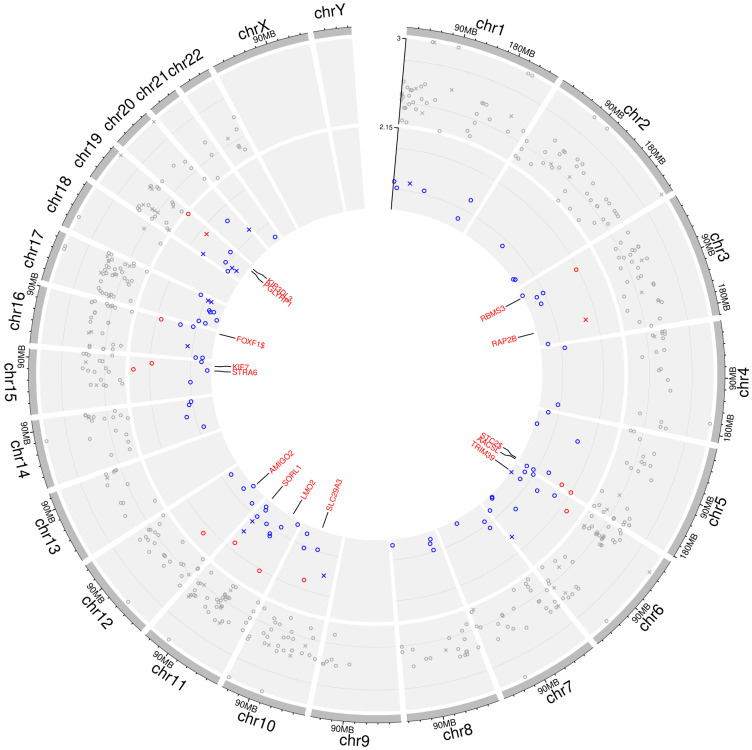
Circos plot of mapped genes for 502 identified CpGs in IOWF2 with statistically significant DNAm × age interaction effects, including those with consistent directions of association in F1 and F2. The circos plot was drawn on the website (https://yimingyu.shinyapps.io/shinycircos/). On the outer track, the points represent −log10 transformed FDR *p*-values of the identified 502 CpGs in IOWF2. Points on the inner track denote −log10 transformed *p*-values for 102 of the 502 CpGs found in IOW (F1), showing consistent direction of associations with those in IOWF2, and red circles highlight the ones with consistent direction of association and being statistically significant (14 CpGs). Each track uses its own data-driven radial (Y-axis) scale, with the outer track ranging from 1.31 (minimum) to 3 (maximum) and the inner track ranging from 0.004 (minimum) to 2.15 (maximum). In both the outer and inner tracks, the cross (×) symbols indicate CpGs corresponding to genes identified in the significant biological pathway (GO:0001775). Chromosome numbers are shown on the outermost circle. Note: “$” in a gene name in the circos plot denotes that the name is from the UCSC genome browser (https://genome.ucsc.edu/).

Using the *ttscreening* package, we identified 601 CpGs showing potential association with SPT at one year of age (Supplemental Table S1). We analyzed biological pathways associated with the genes (429 genes in total) mapped to these 601 CpGs. One significant biological process, cell activation (GO:0001775), was identified at FDR = 0.05 (raw *p*-value = 7.45 × 10^−7^ and FDR-adjusted *p*-value = 0.017; Supplemental Table S2 and Figure S1 in the Supplemental Material for the top 20 pathways with the smallest FDR *p*-values). A total of 53 genes were included in this biological process, and 60 of the 601 CpGs were mapped to these 53 genes (marked as “cross” in the outer circle of Figure 1; Supplemental Table S1).

Each of these 601 CpGs was then included in a logistic regression model with repeated measures to assess its age-specific association with SPT, adjusting for potential confounding factors. At FDR = 0.05, DNAm at 502 CpGs showed statistically significant interaction effects with age (Outer circle of Figure 1; Supplemental Table S3). At 84 (16.7%) of the 502 CpGs, DNAm was negatively associated with the log-odds of SPT at baseline (the higher the DNAm, the lower the log-odds of SPT). However, due to the positive interactions between DNAm and age, such associations were attenuated over time.

Among the 502 CpGs, 111 (22.1%) CpGs were located in the TSS regions, in particular, 66 (13.1%) in the TSS1500 and 45 (9.0%) CpGs in the TSS200 regions. For the remaining identified CpGs, 15 (3.0%) were located in the 1st exon, 21 (4.2%) in the 3′ UTR, 57 (11.4%) in the 5′ UTR, 151 (30.1%) in gene bodies, and 147 (29.3%) in intergenic regions. Notably, 56 (93%) of the 60 CpGs enriched in the cell activation (GO:0001775) process were among the 502 CpGs. When cell-type-adjusted DNAm was analyzed following the same procedure, of the 601 CpG sites that passed the screening, 234 CpG sites showed statistically significant interaction effects between DNAm and age at FDR = 0.05 (Supplemental Table S4). Of these 234 CpGs, 226 (97%) were also present in the cell-type-unadjusted models with consistent directions of association. Pathway analysis using the 234 CpGs also identified the cell activation (GO:0001775) process as the top-ranked pathway, but no longer statistically significant after adjusting for multiple testing by controlling FDR at 0.05 (raw *p*-value 5.36 × 10^−5^ and FDR-adjusted *p*-value = 0.899; Supplemental Table S5). The statistical insignificance was likely due to the reduction of genes in the gene set corresponding to the cell activation process (27 of the 53 genes retained).

### 2.3. Findings in the Replication Cohort, IOWBC (F1)

Of the 502 CpGs showing an interaction effect in IOWF2, consistent directions of association were observed at 102 CpGs in IOWBC (F1) (Supplemental Table S3), of which 14 CpGs were statistically significant (Inner circle of Figure 1; Table 2). Of these 14 CpGs, 6 (43%) were in the promoter region. In addition, the 14 CpGs included those mapped to *PGLYRP1* (cg08693172) and *RAP2B* (cg22678398) genes, both of which were involved in the identified cell activation process (GO:0001775). When using cell-type-adjusted DNAm in the models, of the 234 CpGs showing an interaction effect in IOWF2, consistent directions of association were observed at 99 CpGs, with 10 CpGs being statistically significant. Of these 10 CpGs, 9 were among the aforementioned 14 CpGs.

### 2.4. Gene Expression and MethQTL Analysis

To assess the biological relevance of the replicated 14 CpGs, we evaluated the association of DNAm at these CpG sites with expression of their mapped genes in IOWF2. DNAm at four CpGs showed a statistically significant association with the expression of their respective genes (Table 3, Figure S2 in the Supplemental Material). Two CpGs, cg09089242 (*LMO2*) and cg11606444 (*SORL1*) on chromosome 11, showed negative associations (the higher the DNAm, the lower the expression). In contrast, DNAm at cg21566177 (*STC2*) and cg25314111 (*AACS*) on chromosome 5 had positive associations, with the strongest association observed at cg25314111 (regression coefficient = 0.547).

Among the 14 identified CpGs in both cohorts, 8 CpGs (57%) were identified as methQTL sites (Table 3), where DNA methylation was significantly associated with nearby genetic variants (SNPs) (Supplemental Table S6). The proportion of DNAm variance explained by the lead SNP ranged from 4.328% for cg01519508 (rs923631) to 21.707% for cg21566177 (rs72810976), suggesting a partial influence of underlying genetic variation at these loci. Of the 8 methQTL sites identified in our analysis, 7 (87.5%) were also reported in the GoDMC database [13], supporting the robustness of these associations.

## 3. Discussion

In this epigenome-scale longitudinal study, we explored age-specific associations between DNAm at birth and childhood atopy based on data from two cohorts: the IOWF2 as the discovery cohort and the IOWBC (F1) as the replication cohort. Age-specific association was observed at 502 CpGs in IOWF2, of which 102 (20%) CpGs showed consistent directions of interaction effects in the IOWBC (F1), with 14 CpGs being statistically significant. Of these 14 CpGs, eight (57%) were methQTLs.

Among the 14 identified CpG sites, except for cg01519508 (*FOXF1*), all other CpGs exhibited positive associations with the odds of SPT at one year of age (baseline); a higher DNAm is associated with a higher odds of SPT. However, such positive associations were attenuated over time, indicating a decline in their potential risk effects as children grew. DNAm at cg01519508 showed an inverse association with SPT status at baseline (log odds = −2.436, *p* = 0.008), indicating that higher DNAm at this site was protective early in life. However, a positive DNAm × time interaction effect (log odds = 0.408, *p* = 0.009) suggested that this protective effect diminished as children grew older.

The identified biological process (cell activation) is essential to immune function and plays a critical role in the pathogenesis of atopic diseases, including asthma and allergies [14,15]. For example, the activation of T helper 2 (Th2) cells drives the production of cytokines such as IL-4, IL-5, and IL-13, which promote IgE production and eosinophil activation, key events in allergic inflammation [16]. Similarly, mast cell activation through the high-affinity IgE receptor exacerbates allergic responses by releasing histamine and pro-inflammatory cytokines [17]. Among the 53 genes enriched in this biological process, several play important roles in the development of allergic diseases, e.g., *CD300A*, *CHD7*, and *CNR2* have been linked to allergic inflammation, atopy, and immune regulation [18,19,20].

DNAm at birth at four CpGs was associated with expression of their corresponding genes, suggesting a possible link between early life epigenetic marks and allergic sensitization. Among these, methylation at cg09089242 (*LMO2*) and cg11606444 (*SORL1*) was associated with reduced gene expression. *LMO2* is a transcription factor important for the development of hematopoietic stem cells and T-cell differentiation [21,22], and its expression in cord blood, together with its role in early hematopoiesis, underscores the biological relevance of this finding. Suppression of *LMO2* expression through higher methylation may be related to changes in lymphoid lineage proliferation or immune cell differentiation, potentially influencing immune responses relevant to allergic diseases. Similarly, *SORL1* regulates lysosomal function and receptor trafficking [23,24], and its reduced expression may be associated with modulation of immune or inflammatory pathways. In contrast, methylation at cg21566177 (*STC2*) and cg25314111 (*AACS*) was associated with increased gene expression, and both CpGs were identified as methQTLs, with R^2^ values of 21.707% for *STC2* and 7.725% for *AACS* based on lead SNPs. *STC2* encodes a secreted glycoprotein involved in anti-inflammatory regulation and protection against oxidative stress [25,26], while *AACS* is involved in ketone body and lipid metabolism [27], which are implicated in immune and inflammatory regulation [28,29].

The lack of DNAm–expression associations for the remaining CpGs may be due to tissue or cell-type-specific expression, which may not be fully captured in cord blood. In addition, DNAm at some CpGs may regulate distal genes rather than their annotated genes or may influence biological function through mechanisms other than gene expression, such as regulation of alternative splicing via chromatin-mediated processes [30].

The eight methQTL CpGs indicate that genetic variation may contribute to DNAm variation at these sites. However, given the relatively low R^2^ values observed in the methQTL analyses with the lowest of 4.328% (cg01519508) and the highest of 21.707% (cg21566177) based on lead SNPs, genetic contributions to DNAm variation at these loci are likely to be limited. It is also possible that strong methQTL SNPs were not captured due to limited genotyping coverage or the design of our methQTL analysis, which focused on SNPs located on the same chromosome as the CpG site of interest.

To our knowledge, this is the first study to examine how neonatal DNAm patterns are associated with childhood allergic sensitization status over time. An important strength of this study is its longitudinal design, in which DNAm measurement at birth precedes SPT measurement, reducing the risk of temporal ambiguity or reverse causation. By leveraging repeated measures, we gained higher power to detect changes over time and individual differences using a time-lagged approach. In addition, the two-stage analytical strategy, with screening potentially informative CpGs as the first step, also contributed to the improvement of statistical power. Unlike many epigenetic studies, our parallel analyses with and without cell-type adjustment indicated that findings, including enrichment of the ‘cell activation’ pathway, largely agree with those obtained with adjustment for cell-type heterogeneity. Although the number of significant CpGs reduced after adjustment, the persistent findings suggest that these signals may not be solely driven by cell composition.

As with other epidemiological studies, the present study has some limitations. One such limitation is the utilization of IOWBC (F1) as a replication cohort for the findings in IOWF2, the discovery cohort. Although for almost all F2 participants, one parent was outside of the F1 replication cohort, F1 and F2 are not fully independent. Validating the findings in an independent cohort with similar time points is certainly desired to ensure that results replicated in F1 are valid, given the partial overlap between the two generations. However, one needs to be cautious when applying the findings to general populations, especially for the eight CpGs that are shown to be methQTLs. In the F2 generation, for some subjects, DNA methylation was derived from cord blood samples, and for others from Guthrie cards, whereas in the F1 generation, Guthrie cards were used for all the subjects. Although it has been shown that, on average, DNA methylation at about 70% CpG sites agrees between the two tissues [31], the ~30% inconsistency on average and variations in each source might have caused large variations in the estimates, leading to inconsistent findings. Other factors that possibly affected the results include methylation platforms (Illumina 450K vs. EPIC arrays) and timing of SPT assessment (early childhood vs. later childhood/adolescence). It is also worth noting that the focus of the study was to examine the temporal effects of potentially informative CpGs identified at baseline (one year of age); therefore, CpGs were screened based on SPT status at one year. However, by doing so, we might have missed CpGs showing associations with atopic status at later ages. The identified biological process (cell activation) and its close connection with the development of atopy imply that DNA methylation in blood is nevertheless informative with respect to the underlying epigenetic mechanism of atopy. More importantly, our identified CpGs have the potential to serve as birth markers for future atopic status and are beneficial to the prediction and prevention of childhood atopy that can start soon after birth.

## 4. Materials and Methods

### 4.1. Discovery Cohort—The Isle of Wight Third-Generation Birth Cohort (IOWF2)

The discovery cohort in this study was the IOWF2 generation (children) of a prospective birth cohort on the Isle of Wight (IOWBC; F1 generation) in the United Kingdom. The IOWBC (F1) was established in 1989, focusing on studying the natural history of asthma and allergic disorders [32]. Children in the F2 generation (IOWF2) were born between 2010 and 2022 (*n* = 611), with at least one of the two parents being a participant in the IOWBC (F1) [33]. The International Study of Asthma and Allergy in Childhood (ISAAC) questionnaires were administered at one year, three years, and six years of age. In total, 299 IOWF2 participants had both DNAm and SPT data. The study was approved by the Local Research Ethics Committee. Informed written consent was obtained from the parents of the participants at recruitment.

### 4.2. Outcome Variable: Atopy

Atopy or allergic sensitization was assessed by skin prick test (SPT) at ages 1, 3, and 6 years for 11 common allergens (house dust mite, cat dander, dog dander, grass pollen, tree pollen, Alternaria, cow’s milk, egg, peanut, sesame, and cod). A wheal size ≥ 3 mm above the control was regarded as positive, as previously described [34].

### 4.3. Independent Variable: DNA Methylation

DNA was extracted at birth from 192 participants’ umbilical cord blood and 107 participants’ blood spots on Guthrie cards via a standard salting out procedure [35]. DNAm was measured by the Illumina Infinium MethylationEPIC BeadChips or Human Methylation450 BeadChip (San Diego, CA, USA). Details of DNAm assessment and preprocessing were in Supplemental Material S1.

### 4.4. Cell-Type Composition

DNAm measured in blood may be influenced by variations in cell composition. To account for this, cell-type compositions were estimated using the *estimateCellCounts* function in the R package *minfi* [36]. For cord blood, proportions were estimated for B cells, CD4+ T cells, CD8+ T cells, granulocytes, monocytes, natural killer (NK) cells, and nucleated red blood cells [37]. For Guthrie cards, proportions of B cells, CD4+ T cells, CD8+ T cells, eosinophils, monocytes, NK cells, and neutrophils were estimated [38].

To evaluate the influence of cell-type compositions on the association between DNAm and atopic status, analyses were conducted using cell-type-adjusted DNAm and cell-type-unadjusted DNAm, separately. Throughout, for cell-type-unadjusted DNAm, “DNAm” is used for simplicity and fully spelled out for cell-type-adjusted DNAm. Cell-type-adjusted DNAm values were derived by regressing M-values against cell-type compositions specific to each blood source, and the residuals from these models were used as the adjusted DNAm values.

### 4.5. Confounders

Confounders were selected based on their potential association with DNAm and atopy. The confounders were birth weight, gender, and age [11,12,39]. Information regarding gender and birth weight was collected at birth from hospital maternity records. Age was recorded at the time of SPT data collection (1, 3, and 6 years).

### 4.6. Gene Expression

Gene expression at birth was assessed using total RNA extracted from umbilical cord blood in the F2 generation, collected in PAXgene Bone Marrow RNA tubes (Qiagen, Valencia, CA, USA), following the manufacturer’s protocol. Assessment of total RNA yield and DNA contamination was performed using a Qubit 2.0 fluorometer (Life Technologies, Grand Island, NY, USA). RNA quality was assessed by using Agilent 2100 Bioanalyzer with RNA 6000 Nano Chips (Agilent Technologies, Santa Clara, CA, USA) to determine the 28S/18S rRNA ratio and RNA Integrity Number (RIN). Only samples with RIN values greater than 8260/280 absorbance ratios above 1.8 and 260/230 ratios above 1.5 were included for microarray analysis. Details of gene expression assessment and quality control are described elsewhere [40]. For the expression values, log_2_-transformed intensities normalized to the 75th percentile across all samples were reported.

### 4.7. Replication Cohort—The Isle of Wight Second (F1) Generation Birth Cohort (IOWBC)

For the replication study, the F1 generation of the cohort (parents of the IOWF2), consisting of 1456 individuals born between January 1989 and February 1990 (after exclusion of adoptions, infant deaths, and denials), was recruited [32]. SPT was conducted at ages 4, 10, and 18 years. For ages 10 and 18 years, allergic sensitization status to each of the 11 allergens was available except for sesame, which was unavailable at all ages. Sensitization status to the tree was unavailable at age 4 years. To assess the impact of this allergen on the determination of atopic status, a Chi-square test at age 10 was conducted with and without the tree allergen to evaluate the sensitivity of atopy to tree-related data. The results indicated no difference in terms of the status of a child being atopic.

In the F1 generation, DNAm at birth was measured in DNA extracted from Guthrie cards using Illumina Infinium MethylationEPIC BeadChip (Illumina, Inc., San Diego, CA, USA). DNAm of 717 participants was used to replicate findings in the F2 generation. Cell-type compositions of B cells, CD4+ T cells, CD8+ T cells, eosinophils, monocytes, NK cells, and neutrophils were estimated using the EpiDISH algorithm [41] and used to calculate cell-type-adjusted DNAm.

### 4.8. Statistical Analysis

**Figure 2:**
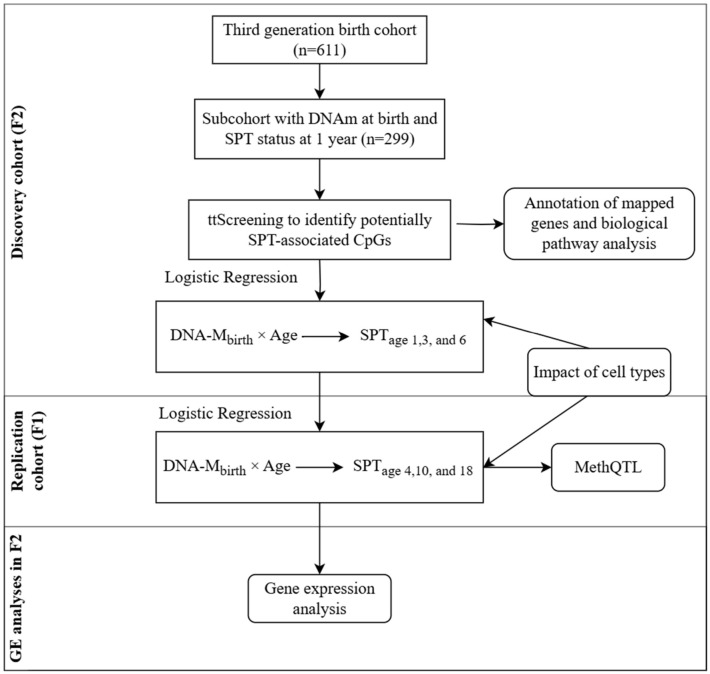
Analytic process of epigenome-scale longitudinal study on SPT with DNAm at birth. Covariates included in the analyses were birth weight, age, and sex of the child.

The workflow of the analytical plan is shown in Figure 2, with the details of each step provided below.

#### 4.8.1. Descriptive Analyses

To determine whether the participants in the study reasonably represented the complete IOWF2 cohort, the demographic characteristics and clinical outcomes were summarized and reported. Continuous variables were analyzed using one-sample *t*-tests, while categorical variables were evaluated using one-sample proportion tests.

#### 4.8.2. Screening of CpG Sites and Analysis of Longitudinal Association

In this epigenome-wide association study, to identify CpGs with DNAm associated with longitudinal SPT outcomes, a two-step approach was employed to improve statistical power: (1) screening for potentially informative CpGs with respect to SPT at age one year, and (2) applying statistical analyses to each potentially informative CpG to assess its association with SPT longitudinally after adjustment for potential confounders. Details of the two steps are in Supplemental Material S2. Briefly, in the first step, we focused on detecting CpGs based on SPT status at one year of age, using the *ttScreening* package in R [42]. CpGs that passed screening were deemed as potentially informative CpGs at baseline. These CpGs were identified through training and testing data with DNAm at birth as the outcome variable and atopic status as the independent variable. Due to the time order, in this screening, we did not adjust the effects of unknown factors (estimated by surrogate variables) and cell-type compositions but focused on differences in DNAm between atopic status (and thus potentially informative CpGs). In the second step, logistic regression with repeated measures was applied with DNAm at each CpG site as the independent variable and SPT status at ages 1, 3, and 6 years as the outcome variable. For the identified CpGs, sensitivity analyses were conducted to evaluate the impact of cell-type composition.

#### 4.8.3. Replication Analysis

The identified CpGs were further examined in the parental cohort (F1) for the consistency of results observed in F2. Logistic regression with repeated measures was applied to assess the associations of the CpGs identified in F2 with SPT at ages 4, 10, and 18 years. We included the same confounders as those for F2 in the regression models. Sensitivity analyses on the impact of cell composition on the associations between DNAm and SPT were conducted in the same manner as in F2. Statistical significance was set at 0.05 for this replication analysis.

#### 4.8.4. Biological Pathways

Pathways and biological processes involving genes to which CpGs passing the screening were mapped would provide valuable insights into the function of those genes. While cell-type adjustment is important for controlling confounding, it may also remove biologically relevant variation in certain contexts, particularly in exploratory functional analyses. Considering this, the cell-type-unadjusted screened CpGs were used to explore broad biologically relevant processes. In addition, a secondary pathway analysis was conducted using the CpGs obtained from the cell-type-adjusted analysis to evaluate whether pathway enrichment results were consistent after accounting for cell-type composition. Gene mapping was based on Illumina manifest annotations, including gene location and chromosomal information. Pathway enrichment analysis was conducted using the *gometh* function in the R package *missMethyl* [43], a method specifically designed for Illumina methylation array data. This approach accounts for the unequal distribution of CpG sites across the genome and adjusts for CpGs mapped to multiple genes. *p*-values for each gene ontology category were calculated using Wallenius’ noncentral hypergeometric test to address potential bias, and multiple testing correction was applied by controlling FDR at 0.05.

#### 4.8.5. Association Between DNAm of Identified CpGs and Expression of Their Mapped Genes

For the CpGs identified in both discovery (F2) and replication (F1) cohorts, to evaluate the potential regulatory functionality of these CpGs on genes, the association between DNAm at these CpGs and the expression of their corresponding mapped genes was examined using data in F2 (*n* = 161). Linear regressions were used with gene expression as the outcome, and DNAm as the exposure variable. For genes represented by multiple probes, the estimated association showing the smallest *p*-values was reported and used to represent the strength of epigenetic regulation (regression coefficient and *p*-value) for the corresponding genes. The statistical significance was set at *p*-value < 0.05.

#### 4.8.6. Methylation Quantitative Trait Locus (methQTL) Analysis

Genotype data, including imputed variants, were subjected to standard quality control procedures. SNPs with minor allele frequency (MAF) < 0.01 and Hardy–Weinberg equilibrium *p* < 1 × 10^−6^ were excluded. SNP missingness was assessed using a threshold of 0.05, although no SNPs were removed based on this criterion. Imputed SNPs were aligned to the forward strand and GRCh37 coordinates, and variants with an INFO score > 0.80 were retained.

Using these quality-controlled genotype data, for CpGs replicated in F1, we evaluated their potential as methQTL sites using DNAm and genotype data from the F1 generation. β-values were first logit-transformed to M-values, followed by rank normalization [44]. To evaluate the association between SNP genotype and DNAm at each CpG site, we applied linear regression using the *big_univLinReg* function in the *bigstatsr* R package [45], regressing DNAm on all SNPs located on the same chromosome one at a time. Multiple testing was addressed using the Bonferroni correction, with the significance threshold defined as 0.05 divided by the number of SNPs tested per chromosome. CpGs with at least one significant SNP within ±1 Mb were classified as cis-methQTLs, and those outside this window as trans-methQTLs. The strength of each methQTL was assessed by calculating the R^2^ value, which represents the percentage of variation in DNAm explained by genotype.

## 5. Conclusions

At certain CpGs, DNA methylation at birth is associated with childhood atopic status in an age-specific manner, and for CpGs showing association at an earlier age, such associations tend to attenuate over time. The CpGs showing consistent associations across cohorts offer insight into the influence of epigenetic imprinting at birth on underlying biological mechanisms of childhood atopy and may inform future research on early prediction and prevention of allergic disease development.

## Figures and Tables

**Table 1:** Comparison of the complete cohort (*n* = 611) and sub-cohort of study subjects (*n* = 299) on SPT status and potential risk factors.

Variables	Complete Cohort n (%) or Mean (SD)	Sub-Cohort n (%) or Mean (SD)	*p* Value
**Skin Prick Test**			
One year			0.985
Positive	21 (4.85%)	12 (4.82%)	
Negative	412 (95.15%)	237 (95.18%)	
Three years			0.266
Positive	30 (12.99%)	14 (9.27%)	
Negative	201 (87.01%)	137 (90.73%)	
Six Years			0.723
Positive	45 (20.74%)	33 (19.30%)	
Negative	172 (79.26%)	138 (80.70%)	
**Gender**			0.751
Male	339 (55.85%)	162 (54.73%)	
Female	268 (44.15%)	134 (45.27%)	
**Birth weight (g)**	3378.65	3381.39	0.933
	(624.07)	(348.12)	

**Table 2:** Effects and genetic information of 14 CpGs showing statistically significant interactions with age on SPT in both cohorts, with consistent directions of association between the two cohorts.

CpGs	Chr. No	Gene	Location	F2 Cohort			F1 Cohort	
				Log Odds	*p* Value	FDR P ^@^	Log Odds	*p* Value
cg01519508	16	*FOXF1* ^$^		−2.436	0.008		−0.355	0.26
Interaction				0.408	0.009	0.017	0.042	**0.032**
cg02009358	3	*RBMS3*	Body	5.005	0.001		0.994	0.063
Interaction				−0.662	0.013	0.021	−0.075	**0.027**
cg06270147	10	*SLC29A3*	TSS200	1.754	0.048		0.474	0.094
Interaction				−0.343	0.006	0.014	−0.038	**0.042**
cg08135379	12	*AMIGO2*	TSS1500	2.611	<0.001		0.531	0.087
Interaction				−0.469	<0.001	<0.001	−0.050	**0.015**
cg08693172	19	***PGLYRP1***	TSS200	2.614	<0.001		0.329	0.242
Interaction				−0.448	<0.001	0.007	−0.038	**0.037**
cg09089242	11	*LMO2*	TSS1500	2.724	0.001		0.646	0.016
Interaction				−0.470	0.002	0.009	−0.040	**0.022**
cg11606444	11	*SORL1*	Body	5.207	0.001		0.681	0.305
Interaction				−0.788	0.01	0.018	−0.084	**0.036**
cg12340546	6	*TRIM39*	5′UTR	2.893	<0.001		0.483	0.072
Interaction				−0.455	0.006	0.014	−0.037	**0.016**
cg14299455	19	*KIR3DL3*	TSS1500	1.907	<0.001		0.778	0.018
Interaction				−0.313	0.003	0.01	−0.054	**0.007**
cg19570897	15	*KIF7*	Body	4.047	<0.001		0.471	0.243
Interaction				−0.507	0.007	0.015	−0.054	**0.027**
cg21566177	5	*STC2* ^$^		2.849	0.001		0.347	0.19
Interaction				−0.436	0.003	0.009	−0.033	**0.049**
cg22678398	3	***RAP2B***	1stExon	3.242	<0.001		0.520	0.167
Interaction				−0.464	0.001	0.008	−0.047	**0.047**
cg23887948	15	*STRA6*	5′UTR	2.161	0.001		0.551	0.084
Interaction				−0.424	<0.001	0.007	−0.049	**0.009**
cg25314111	5	*AACSL*	TSS1500	3.622	0.002		1.116	0.003
Interaction				−0.598	0.002	0.009	−0.053	**0.024**

Notes: ^$^ Gene names are from the UCSC genome browser. ^**@**^ Only FDR-adjusted *p*-values were included for interaction effects, since when interaction presents, main effects cannot be interpreted without addressing interactions. In the last column (results from replications), statistically significant interaction effects were indicated by *p*-values in bold font. Genes in bold font are involved in the identified biological pathway (GO:0001775).

**Table 3:** Results of gene expression analysis in the F2 cohort, along with the lead SNP with corresponding R^2^ for each CpG identified in methQTL analysis in the F1 cohort.

Gene Expression in the F2 Cohort						MethQTL Analysis in the F1 Cohort	
CpG	Chromosome	Gene	Location	Coefficient	*p* Value	Lead SNP	R^2^ Value
cg01519508 *	16	*FOXF1* ^$^		−0.161	0.226	rs923631	4.328%
cg02009358	3	*RBMS3*	Body	−0.328	0.375	--	--
cg06270147	10	*SLC29A3*	TSS200	0.027	0.623	--	--
cg08135379 *	12	*AMIGO2*	TSS1500	0.038	0.652	rs79609374	10.060%
cg08693172 *	19	*PGLYRP1*	TSS200	−0.309	0.166	rs35247686	6.370%
cg09089242	11	*LMO2*	TSS1500	−0.354	**0.008**	--	--
cg11606444	11	*SORL1*	Body	−0.720	**0.006**	--	--
cg12340546	6	*TRIM39*	5′UTR	−0.054	0.627	--	--
cg14299455 *	19	*KIR3DL3*	TSS1500	0.158	0.189	19:55222465:GC	11.657%
cg19570897 *	15	*KIF7*	Body	0.159	0.505	rs12906938	7.319%
cg21566177 *	5	*STC2* ^$^		0.463	**0.008**	rs72810976	21.707%
cg22678398	3	*RAP2B*	1stExon	0.187	0.284	--	--
cg23887948 *	15	*STRA6*	5′UTR	−0.225	0.114	rs76336272	10.195%
cg25314111 *	5	*AACS*	TSS1500	0.547	**0.023**	rs4073382	7.725%

Notes: ^$^ Gene names are from the UCSC genome browser. *p* values < 0.05 are in bold font, and CpGs marked with * denote methQTLs.

## Data Availability

Data are available from the corresponding author upon request with justification, in accordance with privacy and ethical restrictions.

## Supplementary Materials

The following supporting information can be downloaded at: https://www.mdpi.com/article/10.3390/epigenomes10020033/s1, Figure S1: Dot plot of the top 20 biological processes; Figure S2: Scatter plots of DNAm at CpGs associated with expression of their mapped genes. Table S1: Ttscreening results for CpGs at birth potentially associated with SPT at 1 year of age in the F2 cohort; Table S2: Top 20 biological pathways identified using the 601 screened CpGs; Table S3: Age-specific associations of DNAm at CpGs with SPT in the F2 cohort and replication in the F1 cohort for CpGs with significant DNAm × age interactions; Table S4: Age-specific DNAm-SPT associations after cell types adjustment in the F2 cohort and replication in the F1 cohort for CpGs with significant DNAm × age interactions; Table S5: Top 20 biological pathways identified using the 234 cell-type-adjusted CpGs; Table S6: MethQTL results for the 14 CpGs identified in both cohorts. References [46,47,48] are cited in the Supplementary Material.
