# Supplementary material for: DNA Methylation at Birth Showing Age-Specific Association with Atopy in Children: A Prospective Longitudinal Study
Source: Epigenomes. 2026 May 27;10(2):33. doi: 10.3390/epigenomes10020033 (PMC13298419; doi:10.3390/epigenomes10020033)
Supplement: Supplementary file 1 [file epigenomes-10-00033-s001.zip › Supplemental material.pdf]

DNA Methylation at Birth Showing Age-Specific Association with Atopy in Children: A Prospective Longitudinal Study

Supplemental Material

Nahid Sultana[1], Fen Yang[1], Negusse Kitaba[2], Stephen Potter[3], John W. Holloway[2], S. Hasan Arshad[3,4], Hongmei Zhang[1*]

1 Division of Epidemiology, Biostatistics, and Environmental Health, School of Public Health, University of Memphis, Memphis, TN, 38152, USA.
2 Human Development and Health, Faculty of Medicine, University of Southampton, Southampton, SO16 6YD, UK.
3 The David Hide Asthma and Allergy Research Centre, Isle of Wight, P030 5TG, UK.
4 Clinical and Experimental Sciences, Faculty of Medicine, University of Southampton, Southampton SO16 6YD, UK.

* Correspondence: Hongmei.Zhang@memphis.edu

S1. DNA methylation assessment and pre-processing

DNA was extracted at birth from 192 participants' umbilical cord blood and 107 participants' blood spots on Guthrie cards via a standard salting out procedure [35]. Per the manufacturer's standard protocol, one microgram of DNA was bisulfite-treated using the EZ 96-DNA methylation kit (Zymo Research, CA, USA) for unmethylated cytosine to thymine conversion. DNAm was measured by the Illumina Infinium Human Methylation450 BeadChip (>484,000 CpGs) or, when available, Illumina Infinium MethylationEPIC BeadChip (>850,000 CpGs) (Illumina, Inc., San Diego, CA, USA). For both platforms, CpGs that did not achieve a detection p-value of 10^{-16} in at least 95% of samples were excluded, as well as CpGs with probe SNPs within 10 base pairs and with a minor allele frequency > 0.007. In addition, CpGs on sex chromosomes were also excluded to avoid potential bias. CpG sites common to both platforms were included in this study. Intensities of DNAm were quantile-normalized using the *minfi* package in R [36]. Methylation levels of each CpG site (β values), ranging from 0 (no cytosine

methylation) to 1 (complete cytosine methylation), were calculated as a ratio of methylated (Me) to the sum of methylated and unmethylated (U) probes ($\beta = Me/(c + Me + U)$), with a constant (c) to avoid division by zero. Batch effects were then corrected using the R package ComBat function in the *SVA* package in R [46]. After pre-processing, 294,265 CpG sites were retained for statistical analyses. M-values calculated as the logit-transformed β-values ($M = \log2 (\beta/1 - \beta)$) were used in subsequent analysis as previously suggested [47].

S2. Screening of CpG sites and analysis of longitudinal association

In this epigenome-wide association study, to identify CpGs with DNAm associated with longitudinal SPT outcomes, a two-step approach was employed to improve statistical power: 1) screening for potentially informative CpGs with respect to SPT, and 2) statistical analyses applied to the CpGs that passed screening to assess their association with SPT longitudinally, with effects of potential confounders adjusted.

In the first step, we focused on detecting potentially informative CpGs based on SPT status at the "baseline", one year of age. Screening in this way allowed us to prospectively examine in the second step how and to what extent the association between DNAm and SPT evolved, i.e., the existence and strength of age-specific DNAm and SPT associations. An R package, *ttScreening*, was applied to carry out the screening. The screening approach implemented in *ttScreening* applies a training (using 2/3 of the sample set) and testing (using 1/3 of the sample set) process using robust regressions to filter out uninformative CpGs. CpGs identified as statistically significant (p-value < 0.05) in the training process were subsequently evaluated in the testing step (p-value < 0.1). This training-testing process was repeated 100 times to ensure stability of selection. CpGs showing statistical significance in at least 50% of

randomly selected training and testing pairs were considered informative and included in subsequent analyses [42].

In the second step, logistic regression with repeated measures was applied using DNAm of each CpG site as the independent variable and repeated measurements of SPT status at ages 1, 3, and 6 years as the outcome variable. The models were adjusted for potential confounders, including age (treated as a continuous variable), gender, and birth weight. Finally, the age-specificity of the association was examined via DNAm × age interactions. PROC GENMOD in SAS 9.4 with a logit link and binomial distribution was used to fit the logistic regression models with repeated measures. Multiple testing was adjusted by controlling the false discovery rate (FDR) at 0.05, and an FDR-adjusted p-value ≤ 0.05 was considered statistically significant for the analyses [48].

S2. Dot plots of the top 20 biological pathways from Gene Ontology Enrichment Analysis

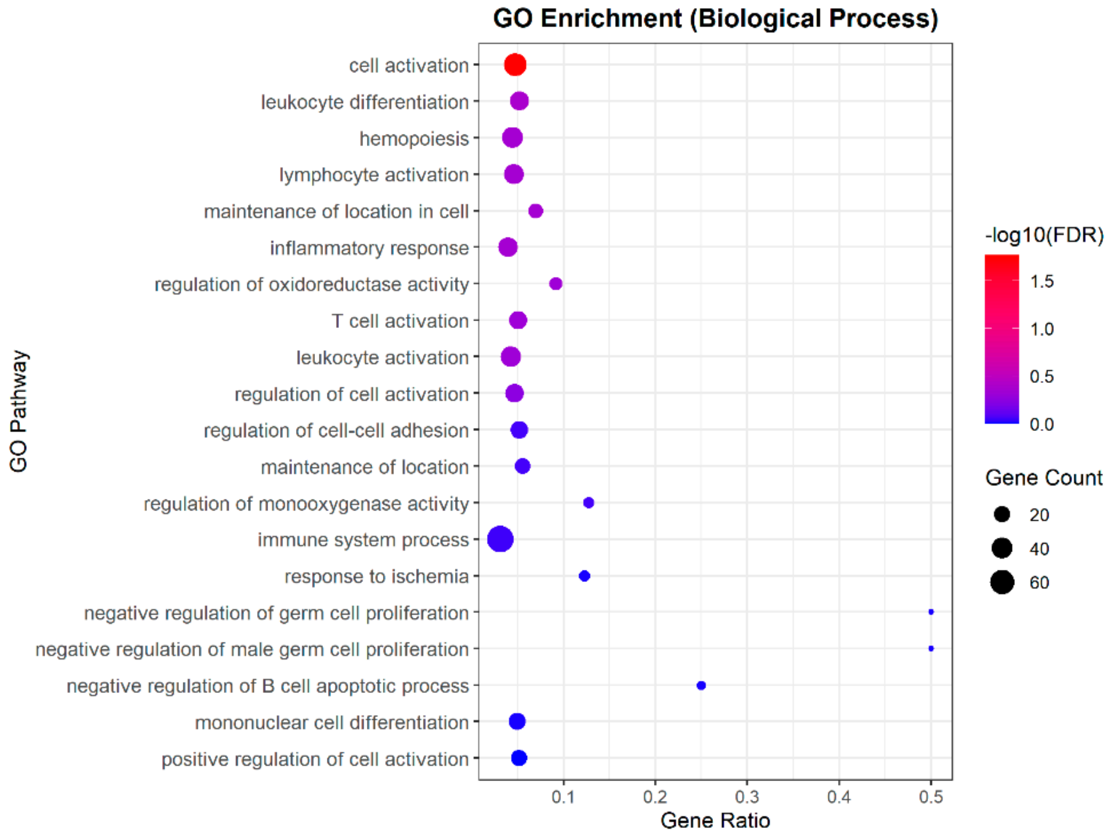

Figure S1. Dot plot (drawn using R package ggplot2) representing the top 20 biological processes, where the x-axis represents the gene ratio (DE/N), with DE being the number of genes mapped from the selected CpGs (601 CpGs) in a given GO term, and N being the total number of genes annotated to that GO term. Dot size corresponds to DE to each pathway, and color represents statistical significance as -log10(FDR), with higher values indicating stronger enrichment. For instance, for the biological process "cell activation", -log10(FDR) is the largest, corresponding to the smallest adjusted p-value (0.017), and the size of the dot suggests it has a relatively large number of genes enriched in this process.

S3. Scatter plots of DNA methylation (in M values) at CpGs associated with expression of their mapped genes

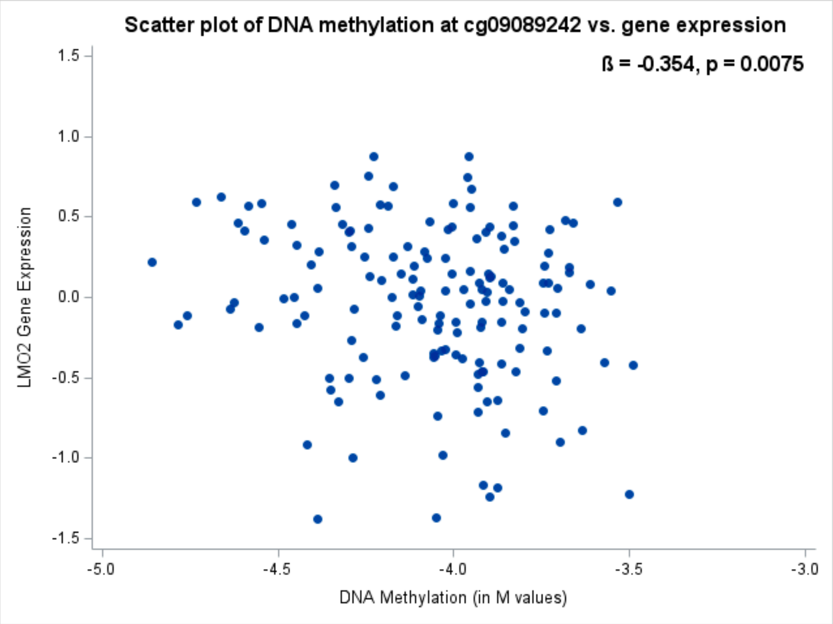
Scatter plot of DNA methylation at cg09089242 vs. gene expression
ß = -0.354, p = 0.0075
LMO2 Gene Expression
1.5
1.0
0.5
0.0
-0.5
-1.0
-1.5
-5.0
-4.5
-4.0
-3.5
-3.0
DNA Methylation (in M values)

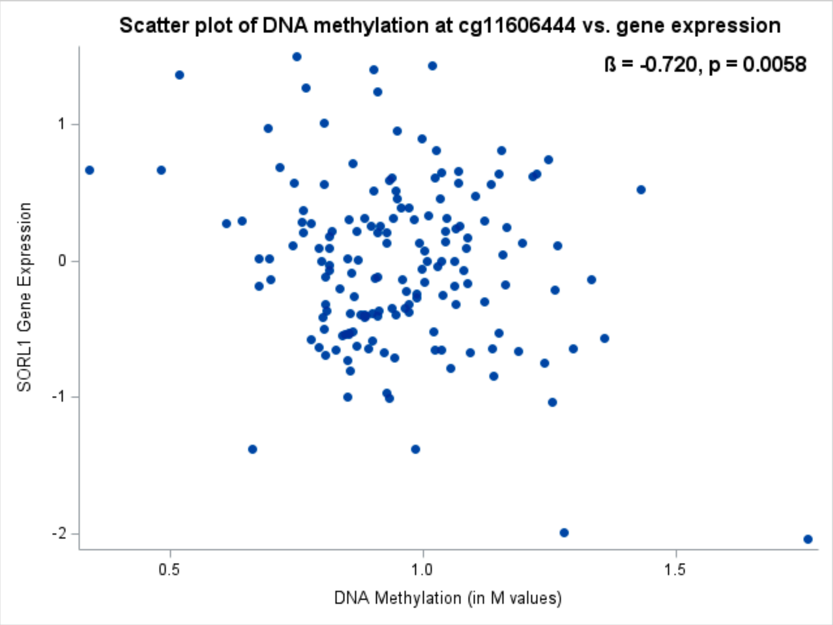
Scatter plot of DNA methylation at cg11606444 vs. gene expression
ß = -0.720, p = 0.0058
SORL1 Gene Expression
1
0
-1
-2
0.5
1.0
1.5
DNA Methylation (in M values)

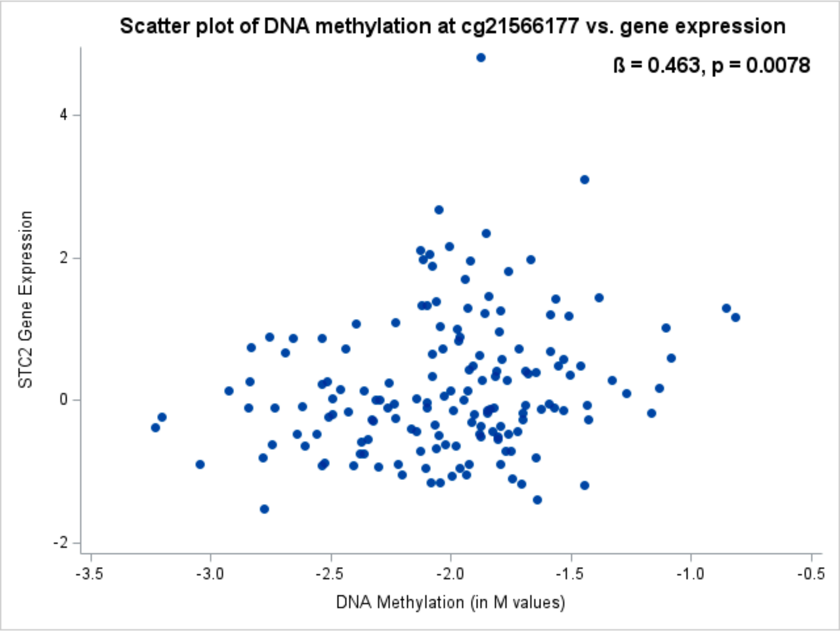

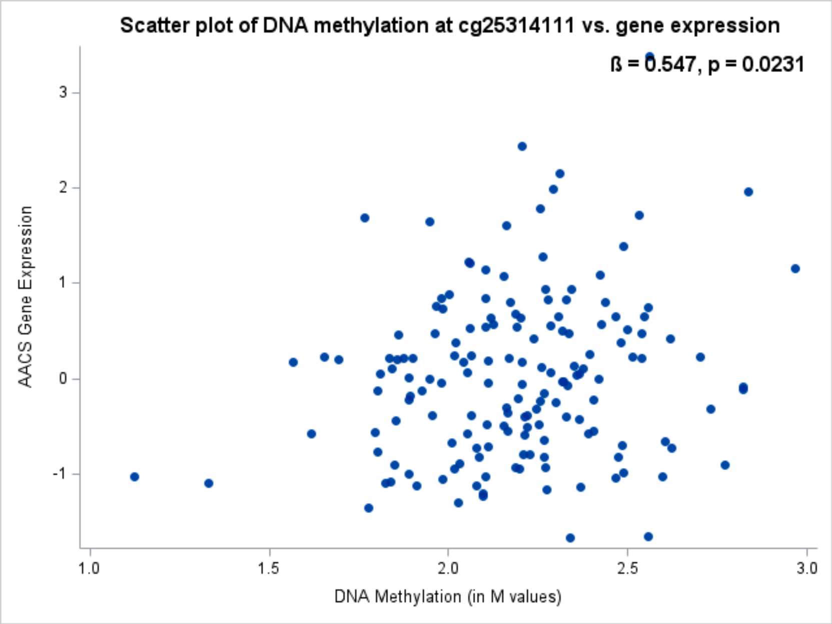

Figure S2. Scatter plots of DNA methylation (in M values) at CpGs associated with expression of their mapped genes.
